# G_2_ chromosomal radiosensitivity in Danish survivors of childhood and adolescent cancer and their offspring

**DOI:** 10.1038/sj.bjc.6602807

**Published:** 2005-10-11

**Authors:** G B Curwen, J F Winther, E J Tawn, V Smart, C A Whitehouse, G S Rees, J H Olsen, P Guldberg, C Rechnitzer, H Schrøder, P E Bryant, X Sheng, H S Lee, R Chakraborty, J D Boice

**Affiliations:** 1 Westlakes Research Institute, Moor Row, Cumbria CA24 3JY, UK; 2 University of St Andrews, St Andrews, Fife KY16 9AJ, UK; 3 Institute of Cancer Epidemiology, Danish Cancer Society, Copenhagen DK-2100, Denmark; 4 Institute of Cancer Biology, Danish Cancer Society, Copenhagen DK-2100, Denmark; 5 The Late Effect Clinic, Juliane Marie Center, Rigshospitalet, Copenhagen University Hospital, Copenhagen DK-2100, Denmark; 6 Department of Pediatrics, University Hospital of Aarhus, Aarhus DK-8200, Denmark; 7 Center for Genome Information, Department of Environmental Health, University of Cincinnati College of Medicine, Cincinnati, OH 45267, USA; 8 International Epidemiology Institute, Rockville, MD 20850, USA; 9 School of Medicine, Vanderbilt University, Nashville, TN 37232, USA

**Keywords:** chromosomal radiosensitivity, childhood and adolescent cancer, inherited predisposition

## Abstract

In order to investigate the relationship between chromosomal radiosensitivity and early-onset cancer, the G_2_ chromosomal radiosensitivity assay was undertaken on a group of 23 Danish survivors of childhood and adolescent cancer, a control group comprising their partners and a group of 38 of their offspring. In addition, the previously reported in-house control group from Westlakes Research Institute (WRI) was extended to 27 individuals. When using the 90th percentile cutoff for the WRI control group, the proportion of individuals with elevated radiosensitivity was 11, 35, 52 and 53% for the WRI control, partner control, cancer survivor and the offspring groups, respectively, with significant differences between the WRI control group and the cancer survivor group (*P*=0.002) and the offspring group (*P*<0.001). However, while the comparisons with the WRI control group support an association of chromosomal radiosensitivity with cancer predisposition, when the partner control group was used to define the radiosensitivity cutoff point, no significant differences in radiosensitivity profiles were found between the partner control group and either the cancer survivor group or the offspring group. The failure to distinguish between the G_2_ aberration profiles of the apparently normal group of partners and the cancer survivor group suggests that any association with cancer should be viewed with caution, but also raises questions as to the suitability of the partners of cancer survivors to act as an appropriate control group. Heritability of the radiosensitive phenotype was examined by segregation analysis of the Danish families and suggested that 67.3% of the phenotypic variance of G_2_ chromosomal radiosensitivity is attributable to a putative major gene locus with dominant effect.

The association between chromosomal radiosensitivity and increased susceptibility to cancer has been demonstrated for a number of cancer-prone heritable conditions, most notably ataxia telangiectasia (Parshad and Sanford, 2001). The cell-cycle-based G_2_ radiosensitivity assay gives the best discrimination and involves irradiating cells *in vitro* during the G_2_ stage of the cell cycle and observing unrepaired damage, in the form of chromatid breaks and gaps, at the subsequent metaphase. The assay was first developed using fibroblasts (Sanford *et al*, 1989) and subsequently applied to lymphocytes (Scott *et al*, 1996). In addition to the rare well-defined cancer susceptibility syndromes, enhanced G_2_ chromosomal radiosensitivity has been demonstrated in a high proportion of unselected breast cancer patients (Scott *et al*, 1994, 1999; Parshad *et al*, 1996; Patel *et al*, 1997; Riches *et al*, 2001), a group of breast cancer patients with a known family history of the disease (Baeyens *et al*, 2002) and a heterogeneous group of adult cancer patients (Terzoudi *et al*, 2000). This led to the suggestion that G_2_ radiosensitivity could be a marker of cancer predisposing genes of low penetrance (Scott *et al*, 1999). Further support for this hypothesis came from a study of first-degree relatives of those with breast cancer, which demonstrated Mendelian heritability of the chromosomal radiosensitivity phenotype (Roberts *et al*, 1999; Scott, 2000). In addition, the proportion of adults showing increased G_2_ chromosomal radiosensitivity has been shown to be higher among those with cancers having an inherited component, such as colon cancer, compared to those with cancers with a predominantly environmental aetiology, such as cervical cancer (Baria *et al*, 2001). In a study of head and neck cancer patients, evidence of increased G_2_ chromosomal radiosensitivity was observed in young patients, thus indicating a genetic contribution to risk for early-onset cases, whereas for older patients, the proportion exhibiting enhanced sensitivity was the same as that for a normal healthy control group (Papworth *et al*, 2001). Using a slightly different technique, chromosomal radiosensitivity has also been reported for patients with glioma and it was suggested that the apparent broad association of mutagen sensitivity with diverse cancers indicates that multiple genes in various DNA repair pathways may be contributing to the integrated phenotype (Bondy *et al*, 2001). Baria *et al* (2002) tested the relationship between chromosomal radiosensitivity and early-onset cancer in a pilot study of patients diagnosed before the age of 20 years and found that 44% of the young cancer patients exhibited increased chromosomal radiosensitivity compared to 15 and 10% of young and adult normals, respectively. This led to the suggestion that a substantial proportion of the patients studied may have a genetic predisposition to cancer mediated through low penetrance genes involved with responding to damage to the genome. However, it was recognised that support for this hypothesis would require the demonstration of heritability of chromosomal radiosensitivity in blood relatives of young cancer patients.

In order to further investigate the heritability of chromosomal radiosensitivity and its relationship with early-onset cancer, we have performed a pilot study using the G_2_ assay to examine the radiosensitivity profile of a group of survivors of childhood and adolescent cancer and their offspring, with the partners of the cancer survivors providing a control group for comparison. In addition, our previously published in-house control group from Westlakes Research Institute (WRI) in Cumbria, UK (Smart *et al*, 2003), was extended by the addition of samples from new individuals.

## MATERIALS AND METHODS

### Study group

Blood samples were obtained from 28 Danish survivors of childhood and adolescent cancer (labelled as T01–T28) who had been treated with radiotherapy, their partners and 44 offspring. This work forms part of a pilot study for the investigation of a range of genetic endpoints associated with germ cell mutagenesis and cancer susceptibility (Boice *et al*, 2003). These former patients were selected from the Danish survivor cohort identified from the files of the nation-wide population-based Danish Cancer Registry. The Danish survivor cohort consists of 4676 cancer survivors diagnosed with cancer under 20 years of age between 1943 and 1996 who lived to be at least 15 years of age. Patients included in the cohort were those alive or born after 1 April 1968 when the national Central Population Register (CPR) was established with personal identification numbers for all citizens, thus permitting linkage among registers. Through linkage to the CPR containing updated information on vital status, migration and first-degree relatives, all partners and liveborn children of eligible survivors were identified. No attempt was made to identify an offspring control group, nor to distinguish between the sexes, since previous studies have found no correlation between induced aberration yields and donor age, or any influence of gender (Baria *et al*, 2002). Approval for the study was obtained from the Danish Scientific Ethical Committee and the Danish Data Protection Agency.

Eligible survivors were contacted by letter to determine their willingness to participate in the study. The survivors and their families were invited to the State Hospital, Rigshospitalet, Copenhagen (for those living in the eastern part of Denmark) or the Skejby Hospital, Aarhus (for those living in the western part of Denmark) to complete a short questionnaire and to have blood drawn. Each of the 28 families who agreed to participate gave informed consent. Detailed information on cancer type was abstracted from medical records. To ensure anonymity, each family was assigned a study number and this was used for all blood samples and questionnaires. The blood samples were further coded to avoid identification of cancer survivor, partner and offspring within each family group. Blood samples were drawn from participating families on Monday and the samples transported directly from Copenhagen to WRI by courier, arriving early Tuesday morning. Samples were hand inspected at customs and, to ensure they had not been exposed to X-rays, a piece of dental film was included with each batch. A total of 100 blood samples were collected and sent to the UK in 10 shipments over 13 months between March 2002 and April 2003.

Each shipment also contained a blood sample from one healthy adult Danish volunteer to act as the control for the transportation system. This volunteer remained the same throughout the study. In addition, repeat blood samples from one healthy adult WRI volunteer were obtained in parallel for seven of the shipments to act as the internal assay control. Some of the samples received at shipment eight failed to culture successfully and this resulted in three families (T16–T18) and the associated transport and internal assay controls being excluded from the study. A further family (T11) was excluded since offspring samples were not available and another (T14) because the final diagnosis of the survivor was not cancer. Thus, only 23 out of the 28 Danish cancer survivor families proved suitable for analysis, that is, a total of 84 samples, together with the nine samples from the transport control and the six samples from the internal assay control. Minisatellite analysis on four stable loci was undertaken to confirm biological paternity and maternity for all the offspring samples collected.

In addition to the cancer survivor families, one sample from each of eight additional healthy control volunteers was taken at WRI to increase the previously reported WRI control group of 19 individuals (Smart *et al*, 2003) to 27 individuals, a number more comparable with that of the cancer survivor group.

### The G_2_ assay

The G_2_ assay was performed according to the method previously described by Smart *et al* (2003), which was based on modified versions of the work performed by Sanford *et al* (1989) and Scott *et al* (1999). Peripheral blood was drawn into lithium heparin vacutainers and cultures (2 ml blood) were set up in prewarmed (37°C) and pregassed (5% CO_2_/95% air) RPMI medium supplemented with 15% foetal calf serum, 1% L-glutamine and 1% phytohaemagglutinin (M-form) to a total volume of 20 ml. Each culture was set up in triplicate, one flask for the determination of the spontaneous aberration frequency and two flasks to be irradiated for the determination of the induced aberration frequency. A single foetal calf serum batch was used for the entire period of the study. Flasks were placed upright in a humidified, CO_2_-gassed incubator. After 48 h, 15 ml of medium was carefully removed and replaced with 15 ml of fresh pregassed, prewarmed medium. At 72 h, the cells were transported in a portable incubator at 37°C and irradiated (or sham irradiated) with 0.5 Gy, 300 kV X-rays. Following a 30 min recovery time, 0.2 ml colcemid (10 *μ*g ml^−1^) was added, and at 90 min after irradiation, the contents of the culture flasks were transferred to centrifuge tubes and plunged into ice chippings. Subsequent centrifugation, hypotonic treatment (0.075 M KCl) and fixation (methanol : glacial acetic acid, 3 : 1) were carried out at 4°C. Fixed cells were stored at −20°C overnight, or longer, prior to slide making.

Metaphase slides were made according to standard procedures and stained with Giemsa. Slides from the family samples together with those from the transport and internal assay control samples were further coded prior to cytogenetic analysis. The eight additional WRI control samples were analysed in conjunction with another ongoing study according to the same criteria. For each irradiated and unirradiated sample, 100 well-spread metaphases were analysed. Chromatid-type aberrations were scored according to previously outlined criteria (Smart *et al*, 2003), with breaks being defined as misaligned discontinuities and gaps as single aligned discontinuities wider than the width of a chromatid. For each irradiated sample, the total number of chromatid gaps and breaks was determined and the spontaneous aberration yield subtracted to give the induced G_2_ aberration yield. Two scorers were used to eliminate scorer bias, with each scorer analysing 50 metaphases per sample.

### Statistical methods

The distributions of aberrations among metaphase cells were analysed for approximation to the Poisson distribution and standard errors were calculated taking into account overdispersion as described previously (Smart *et al*, 2003). For the WRI control group, repeat sampling had been undertaken on nine of the previously reported 19 individuals (Smart *et al*, 2003), and for the calculation of the group mean, individual means were used together with single sample results of the remaining 10 and additional eight new samples. The standard error for this group was calculated adjusting for overdispersion plus the additional intraindividual variation. Aberration frequencies were tested for heterogeneity using the *χ*^2^ test. The nonparametric Mann–Whitney *U*-test was used to compare median induced aberration frequencies between controls and the other donor groups. The 90th percentile values for induced aberrations per 100 cells were calculated for the WRI control group and the partner control group and used as the cutoff points for determining the proportions of radiosensitive individuals in the other groups. Using the WRI control group value, Fisher's exact tests were used to compare the proportion of radiosensitive individuals in the WRI control group with each of the three family groups. Similarly, using the 90th percentile for the partner group, comparisons were made with the cancer survivor group and the offspring group.

The segregation analysis was performed by using the Statistical Analysis for Genetic Epidemiology software (SAGE, 2004). This programme uses a regressive model, in which distribution of the residuals from the genotype means follow multivariate normality (after Box–Cox transformation). In constructing pedigree likelihood, the parent–offspring relationship is explained in the transmission parameter (*τ*_g_), which is the probability that a parent of genotype *g* transmits allele *A* to his/her offspring. We constructed different disease transmission models by constraining *τ*_g_: general model (0⩽*τ*_AA_, *τ*_AB_, *τ*_BB_⩽1), Mendelian model (0⩽*τ*_AA_=1, *τ*_AB_=0.5, *τ*_BB_=0) and sporadic model (*τ*_AA_=*τ*_AB_=*τ*_BB_). The Mendelian model was accepted when (1) the general model did not show a significantly better fit to the data than the Mendelian model and (2) the general model fitted significantly better than the sporadic model. The Akaike information criterion (AIC) (Akaike, 1974) was used to compare the models. In addition to the sex covariate (0 for male, 1 for female), residual correlations from the family members were also included in the model such that residual polygenic effects can be tested (0⩽*ρ*_po_, *ρ*_ss_⩽1, where *ρ*_po_ is correlation between parent–offspring pairs and *ρ*_ss_ is correlation between siblings). For the most parsimonious model, the contribution of the genetic factor to the total phenotypic variance was computed from the ratio of genetic variance (i.e., variability of genotypic means across the putative genotypes) to the total phenotypic variance (an indicator of the heritability of radiosensitivity, attributed to the putative genetic factor inferred by the segregation analysis).

## RESULTS

The radiation-induced G_2_ aberration frequencies for the transport and internal assay controls are shown in Table 1. Analysis of the transport samples indicated a significant intraindividual variation in induced aberration yields (*χ*^2^_8_=24.06, *P*=0.002). The Danish volunteer who provided these samples became pregnant during the course of the study and was pregnant at sample shipments 6–10. Analysis of the samples provided prepregnancy (shipments 1–5) revealed no significant intraindividual variation (*χ*^2^_4_=6.70, *P*=0.153) and a mean induced aberration frequency of 81.00±3.17 per 100 cells, while for those provided during pregnancy, there was a statistically significant difference (*χ*^2^_3_=14.39, *P*=0.002) and a mean induced aberration frequency of 95.50±6.71 per 100 cells. No significant difference was found between the induced aberration frequencies for the samples provided by the internal assay control (*χ*^2^_5_=5.91, *P*=0.315), thus indicating that there was no intrinsic assay effect.

Details of the cancer survivors together with individual radiation-induced G_2_ aberration frequencies for all family members are given in Table 2. Group information on the donor characteristics and the induced aberration frequencies for the WRI controls, partner controls, cancer survivors and the offspring are provided in Table 3. Standardisation of the G_2_ assay had previously been established using 57 blood samples from 19 healthy WRI adult volunteers (Smart *et al*, 2003). The addition of eight samples from new recruits to this group did not significantly affect previous findings. Intraindividual variation was not significant for seven out of nine volunteers (with one of the two that were significant being border-line) and interindividual variation among all 27 individuals remained highly statistically significant (*χ*^2^_26_=60.05, *P*<0.001). Comparison of the median induced aberration frequencies revealed no significant difference between the WRI control and partner control groups, but significant differences were observed when the WRI control group was compared to the cancer survivor group (*P*=0.009) or the offspring group (*P*=0.001). Although the mean values for the partner control group and the cancer survivor group were similar, that for the offspring group was higher (Table 3), this being mainly driven by one high induced yield of 404 aberrations per 100 cells. Confirmation of this unusually high value was obtained by analysing the second irradiated blood sample and a check was made on other samples irradiated at the same time in order to rule out any error in the irradiation procedure.

**Figure 1:**
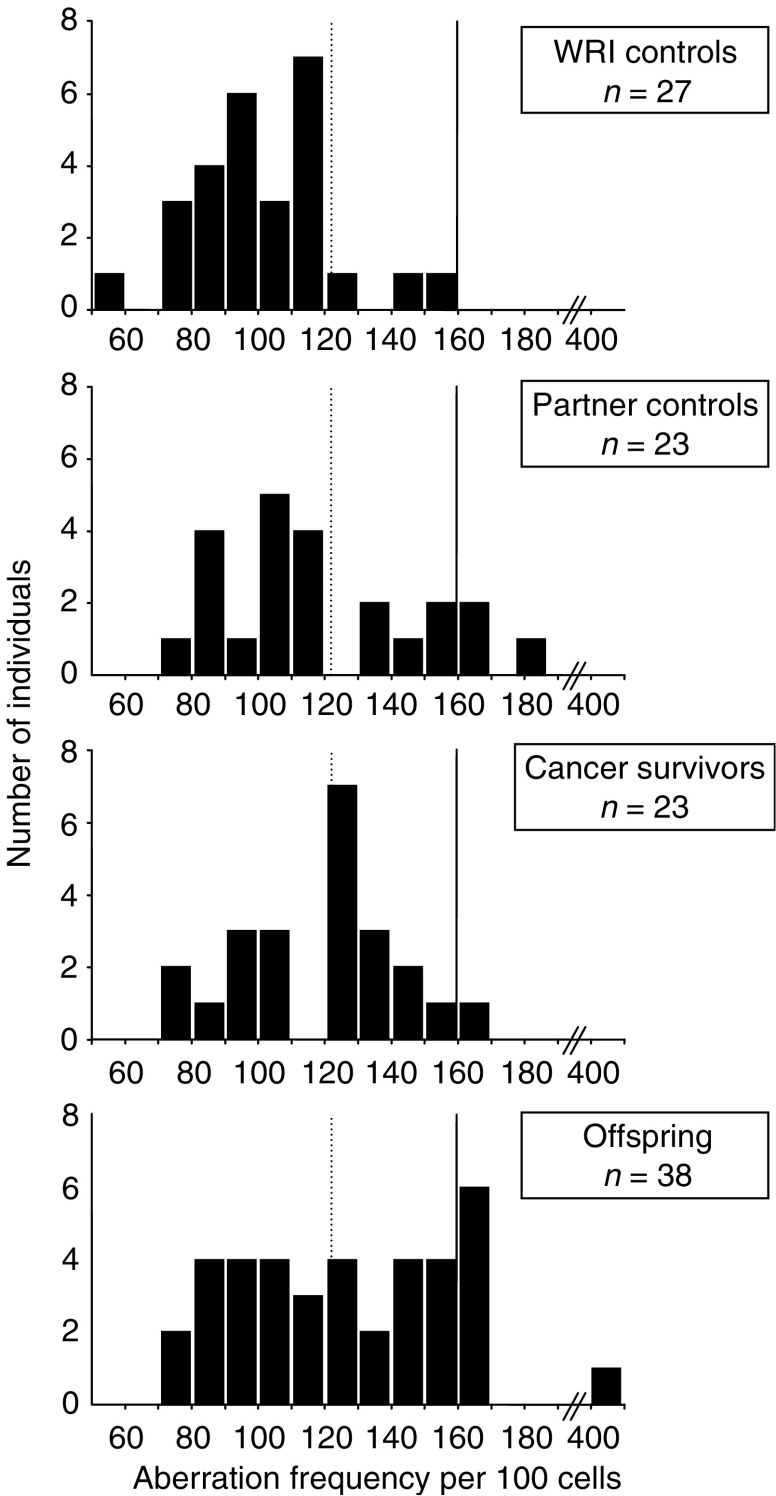
Distributions of G_2_ induced aberration frequencies in four groups of donors. The dotted and solid vertical lines represent the cutoff points for a normal and radiosensitive response, based on the 90th percentile of the WRI control and partner control groups, respectively.

Distributions of aberration frequencies for the WRI control and three family groups are illustrated in Figure 1. Using the 90th percentile cutoff point for the WRI control group of 122 aberrations per 100 cells for a radiosensitive/non-radiosensitive response, the proportion of individuals with elevated G_2_ radiosensitivity was 11, 35, 52 and 53% for the WRI control, partner control, cancer survivor and the offspring groups, respectively. While the difference between the WRI control group and the partner control group did not reach statistical significance (*P*=0.084), significant differences were found between the WRI control group and the cancer survivor group (*P*=0.002) and the WRI control group and the offspring group (*P*<0.001). However, when the 90th percentile value of 160 aberrations per 100 cells for the partner control group was used as the cutoff point for increased radiosensitivity, 13, 4 and 18% of the partner control, cancer survivor and offspring groups, respectively, were classified radiosensitive, while no individual from the WRI control group displayed enhanced radiosensitivity. Using the partner control group to define the radiosensitivity cutoff point resulted in no significant differences in the proportion of sensitive individuals between the partner control group and either the cancer survivor group (*P*=0.608) or the offspring group (*P*=0.729).

Heritability of the radiosensitivity phenotype was examined by segregation analysis (Table 4). The offspring with the high value of 404 aberrations per 100 cells was not included in this analysis. Compared to the general transmission model, the Mendelian model was not rejected (*χ*^2^_3_=1.74, *P*=0.628). The sporadic model with equal transmission probability was rejected by the likelihood ratio test (*χ*^2^_3_=11.6, *P*=0.009). In addition, the Mendelian model showed a smaller AIC value than either the general or the sporadic model. Therefore, the Mendelian model was accepted over the other two models. We further compared the autosomal dominant model with the additive model. Based on the AIC criterion, the autosomal dominant model provided a better fit with a smaller AIC value (779.46). Neither gender covariate (*P*=0.497) nor residual familial correlation (converged into zero) turned out to provide significant effects.

The most parsimonious model (last column of Table 4) predicted from this analysis suggests a major gene locus at which the dominant allele (labelled as *A*) confers a genotypic mean induced frequency of 143.53 aberrations per 100 cells (same for *AA* and *AB* genotypes), with the recessive homozygotes (*BB*) having a mean of 97.82 induced aberrations per 100 cells. With frequencies of 0.450 and 0.550, for the dominant and recessive phenotypes, respectively (shown in the top panel of Table 4), the genetic variance attributable to this putative locus becomes 517.13, with residual variance within each genotype being 250.94 (shown in Table 4). Thus, the estimate of the variance of chromosomal radiosensitivity attributable to the putative dominant major gene locus becomes: 513.13/(513.13+250.94)=0.673, suggesting that more than two-thirds of the phenotypic variance of chromosomal radiosensitivity is attributable to a putative major gene locus (with dominant effect). This heritability estimate is also consistent with an alternative prediction from a variance component analysis of the data of the 23 nuclear families, performed by the SOLAR software (Almasy and Blangero, 1998). For the unadjusted induced G_2_ aberration frequency, the heritability estimate by this variance component method was 0.607±0.215 (significantly different from zero, *P*=0.006), which is not substantially different from the value of 0.670 predicted from the parameter estimates of the model fitting from SAGE.

## DISCUSSION

The G_2_ chromosomal radiosensitivity assay requires stringent technical conditions if reproducible results are to be obtained (Scott *et al*, 1996; Bryant *et al*, 2002). Assay reproducibility has been previously demonstrated in our laboratory (Smart *et al*, 2003) and this had been confirmed in the present study by the analysis of sequential samples from the internal assay control (Table 1). Problems with reproducibility of results associated with storage conditions and transportation of blood samples from distant sources have been reported (Scott *et al*, 1999), although reproducibility has been shown not to be an issue for samples transported over short distances (Roberts *et al*, 1999; Smart *et al*, 2003). In order to minimise any potential effect of transportation, samples were sent to WRI from Denmark in secondary packaging (consisting of a screw-cap polyethylene tube to prevent breakage and contain any spillage), at ambient temperature by road and by air and in the possession of a personal courier at all times. Hence, they were not subjected to any significant temperature fluctuations, a factor that has been suggested as a possible cause of intraindividual variability (Bryant *et al*, 2002). In addition, any potential variation associated with transport was monitored by the inclusion of a sample from a Danish control with each shipment. While the first five samples provided by the Danish control gave no indication of intraindividual variability, which could have been associated with transportation, the final four samples gave variable results (Table 1). However, since the volunteer was pregnant when the last four samples were taken, this variability could be associated with the accompanying hormonal changes. Support for this view comes from *in vitro* studies, which indicate that female reproductive hormones can influence radiation-induced yields of chromosome aberrations (Roberts *et al*, 1997; Ricoul *et al*, 1998), and *in vivo* studies in mice (Ricoul and Dutrillaux, 1991) and humans (Ricoul *et al*, 1997), which report fluctuating radiation-induced chromosome aberration frequencies throughout pregnancy.

Consideration of the three family groups, that is, cancer survivors, their partners and their offspring, revealed no statistical differences in the chromosomal radiosensitivity profiles between the groups (Table 3, Figure 1), and thus, based on the family comparisons, we have not been able to demonstrate an association between increased G_2_ chromosomal radiosensitivity and predisposition to childhood or adolescent cancer. However, when comparisons were made with the WRI control group of healthy volunteers, a clear distinction in radiosensitivity profile is observed for both the cancer survivors and their offspring (Table 3, Figure 1). Using the 90th percentile cutoff for the WRI controls as a measure of enhanced radiosensitivity suggests that 52% of the cancer survivors and 53% of their offspring are exhibiting enhanced sensitivity compared with 11% of the WRI controls. On this basis, our results would seem to confirm previous work on childhood cancer patients (Baria *et al*, 2002). This raises questions as to the suitability of the partners of the cancer survivors to form an appropriate control group. Although no significant differences in either the medians or the percentage of individuals classed as radiosensitive were found between the WRI control and the partner control groups, when using the 90th percentile cutoff for the WRI controls as a measure of enhanced radiosensitivity, 35% of the partner control group were classed as radiosensitive.

Although a transportation effect cannot be ruled out for the enhanced radiosensitivity profiles seen in all three family groups, it is possible that the partners form a distinct group with heightened radiosensitivity. A check of patient and partner questionnaires and cancer registries confirmed that none of the partners had suffered from cancer. However, cancer survivors may be more likely to attract partners with some knowledge of cancer because the partners have experienced it within their own families. Thus, the partner control group may comprise a greater proportion of individuals with cancer predisposition than a control group taken randomly from a healthy population.

One of the offspring had an unusually high value of 404 induced aberrations per 100 cells. To our knowledge, this is substantially greater than any previously reported value for the G_2_ assay using similar techniques. The child was aged 6 years at the time of sampling and is healthy with no history of cancer.

Hodgkin's disease and Wilms’ tumour form the two major disease groups in our study (Table 2). Of the 10 cases of Hodgkin's disease, four exhibited evidence of enhanced radiosensitivity with aberration levels at or above the cutoff point defined by the WRI controls. Similar results were reported by Baria *et al* (2002), who found that three out of six individuals with Hodgkin's disease displayed enhanced radiosensitivity. Three of the six cases of Wilms’ tumour also displayed enhanced radiosensitivity. All six cases were unilateral and for none of them was there any recorded family history of Wilms’ tumour.

Studies of families of patients with breast cancer have provided evidence for the G_2_ chromosomal radiosensitivity phenotype being heritable, since first-degree relatives of patients demonstrating enhanced sensitivity were more likely to be radiosensitive than comparable healthy controls (Roberts *et al*, 1999; Scott, 2000). Segregation analysis of family members suggested that 82% of the variability in G_2_ radiosensitivity could be accounted for by a model with a single major gene with two alleles combining in an additive manner to give three phenotypes (Roberts *et al*, 1999, Scott, 2000). However, although this model explained the majority of the patterns of segregation observed in the breast cancer families, there were a few families for which the inclusion of a second bi-allelic gene gave a better fit to the data.

Our segregation analysis results are different in two respects. Firstly, we found that the one-locus model is most parsimonious (i.e., a second locus is not needed), with alleles having a dominant–recessive relationship with regard to the effect on the induced G_2_ aberration yield. Secondly, the contribution of this locus to the total variance of the G_2_ aberration yield in our data appears to be lower than that estimated by Roberts *et al* (1999). Owing to the limited sample sizes, these differences may not be real, but some methodological differences between the two studies can be recognised. Firstly, unlike our sample of nuclear families, which were not preselected based on G_2_ radiosensitivity, *per se*, Roberts *et al* (1999) include in their analyses families with index cases who showed either most sensitivity or had modal values of G_2_ radiosensitivity. Even though these authors controlled for this ascertainment bias in their pedigree analysis, their single selection method of ascertainment correction may not be adequate (Ewens, 1991). Any unaccounted ascertainment bias due to enrichment by extremely G_2_-sensitive individuals would influence the heritability estimate in an upward direction, and this could account for the difference between their value of 82% and our value of 67% for the proportion of the variance attributed to the putative major gene. Secondly, based on the distribution of G_2_ radiosensitivity of all nonindex cases, Roberts *et al* (1999) used a log-transformation of their original measurements prior to a segregation analysis. In contrast, our unselected families provide a good fit to normality (by a Q–Q plot analysis, data not shown), needing no transformation. The Box–Cox transformation giving the *λ* value of 0.55 (Table 4) was simply to account for comingling of putative differences of three genotype-specific mean values. In contrast, as shown by Hanis and Chakraborty (1984) and Chakraborty and Hanis (1987), when families are preselected based on nonrandom trait values of probands, exclusion of probands alone does not totally account for the bias of distributional properties, and hence, the log-transformation may be another reason for the observed differences of the segregation analyses inference. Thirdly, Roberts *et al* (1999) used the software PAP, and we used SAGE for conducting our analyses, and these two differ with regard to the numerical approximations of likelihood evaluation under the mixed model (i.e., major gene in addition to possible polygenes and environmental effects). Finally, families included in the earlier study had index cases, all of whom had breast cancer, while our index cases (survivors of childhood and adolescent cancer receiving radiation therapy) include a variety of cancers (Table 2). In addition, their suggestion of a putative second locus effect is contingent upon distribution of induced G_2_ aberration yields in offspring of very discordant pairs of spouses, in which the ascertainment bias issue would have been more complex. However, after consideration of these differences and ignoring the finer details, there is agreement between the two studies on the involvement of a single major gene locus, which accounts for over two-thirds of the variation of G_2_ sensitivity, and our study is supportive of genetic heritability of radiosensitivity in families of unselected cancer cases.

In conclusion, with respect to an association between cancer susceptibility and chromosomal radiosensitivity, this study provides inconclusive but intriguing results. It is perhaps worth noting that if reliance had been placed solely on the in-house WRI controls for comparison with the cancer survivors and their offspring, then confirmation of an association between G_2_ radiosensitivity and cancer susceptibility would not be in doubt. However, choosing to study G_2_ radiosensitivity in the partners of the cancer survivors, and finding that this group was statistically indistinguishable from the cancer survivor and offspring groups, has resulted in doubts as to the relevance of the assay as a measure of predisposition to cancer. Nevertheless, this study has provided evidence of heritability of the radiosensitivity phenotype. Future studies will expand all three family groups and also investigate further whether a transport factor could be influencing the results.

## Figures and Tables

**Table 1:** Characteristics of transport and internal assay control donors and G_2_ induced aberration frequencies

			**Induced aberration frequencies per 100 cells at sampling shipments**										
**Donor**	**Sex**	**Age at first sampling (years)**	**1**	**2**	**3**	**4**	**5**	**6**	**7**	**8**	**9**	**10**	**Mean aberration frequency±s.e. per 100 cells**
Transport control	F	37	91	58	87	97	72	98	117	—	110	57	87.44±2.37
Internal assay control	F	37	91	91	—	—	—	109	117	—	109	128	107.50±2.43

s.e.=standard error; F=female; —=data not available.

**Table 2:** Characteristics of childhood and adolescent cancer survivors, their partners and offspring, with G_2_ induced aberration frequencies

**Survivor**						**Partner**		**Offspring^a^**	
	**Gender**	**Primary cancer**	**Age at the time of treatment (years)**	**Age at the time of sampling (years)**	**Aberrations in 100 cells**	**Age at the time of sampling (years)**	**Aberrations in 100 cells**	**Age at the time of sampling (years)**	**Aberrations in 100 cells**
T01	F	Hodgkin's disease	15	35	122	28	84	2	89
								7 mths	146
T02	F	Hodgkin's disease	11	34	125	31	85	3	135
								1	80
T03	M	Rhabdomyosarcoma	9	34	103	32	115	8	91
								5	108
T04	F	Hodgkin's disease	15	36	98	39	162	3	87
		Thyroid cancer^b^	30						
T05	M	Hodgkin's disease	10	36	73	31	153	9	123
								7	139
T06	F	Teratoma	1 mth	33	160	36	114	14	162
								9	156
								6	404
T07	F	Hodgkin's disease	19	29	109	30	100	2	105
T08	M	Neuroblastoma	7 mths	32	134	33	105	2	163
T09	M	Wilms’ tumour	7	33	130	36	112	4	88
								3	97
								1	113
T10	F	Wilms' tumour	4	25	80	26	86	3	114
T12	F	Hodgkin's disease	14	25	128	31	115	11 mths	73
T13	F	Lymphoepithelioma	20	30	124	36	100	4 mths	93
T15	M	Pineocytoma	19	35	98	33	154	7	128
T19	M	Hodgkin's disease	17	35	76	35	132	12	150
								10	96
T20	F	Hodgkin's disease	17	33	146	33	94	3	148
								3	115
T21	F	Hodgkin's disease	19	35	90	37	166	9	151
T22	M	Wilms’ tumour	1	31	130	36	88	2	108
T23	M	Wilms’ tumour	5	32	145	36	101	6	163
								1	140
T24	F	Lymphoblastic lymphoma	14	36	154	37	141	13	124
		Breast cancer^b^	33					9	146
T25	F	Neuroblastoma	1	36	129	37	189	9	159
T26	F	Hodgkin's disease	19	37	120	43	131	13	160
								10	161
T27	F	Wilms' tumour	2	36	104	40	100	7	102
								5	76
T28	F	Wilms' tumour	2	34	121	35	79	3	120
								3	163

F=female; M=male; mths=months.

a Offspring in birth order.

b Secondary cancers.

**Table 3:** Characteristics of WRI control, partner control, cancer survivor and offspring groups, with median and mean G_2_ induced aberration frequencies

**Group**	**Number of individuals**	**Male/ female**	**Median age in years (range)**	**Median aberration frequency per 100 cells (range)**	**Mean aberration frequency±s.e. per 100 cells**
WRI controls	27	11/16	30 (20–54)	95.43 (59–154)	100.39±3.28
Partner controls	23	15/8	35 (26–43)	112.00 (79–189)	117.65±3.00
Cancer survivors	23	8/15	34 (25–37)	122.00 (73–160)	117.35±2.95
Offspring	38	22/16	4.5 (4 mths–14)	123.50 (73–404)	130.95±2.30

WRI=Westlakes Research Institute; s.e.=standard error; mths=months.

**Table 4:** Model parameters from segregation analysis of G_2_ radiosensitivity in 23 nuclear families of survivors of childhood cancer patients

	**Models analysed**				
**Parameter**	**General**	**Equal *τ* (sporadic)**	**Mendelian**	**Autosomal dominant**	**Most parsimonious**
*q_A_*	0.253	0.608	0.280	0.260	0.258
*ψ_AA_*	0.064^a^	0.369^a^	0.079^a^	0.067^a^	0.067^a^
*ψ_AB_*	0.378^a^	0.477^a^	0.403^a^	0.385^a^	0.383^a^
*ψ_BB_*	0.559^a^	0.154^a^	0.518^a^	0.548^a^	0.550^a^
*τ_AA_*	1.000^b^	0.608	1.000^b^	1.000^b^	1.000^b^
*τ_AB_*	0.666	0.608^a^	0.500^b^	0.500^b^	0.500^b^
*τ_BB_*	0.000^b^	0.608^a^	0.000^b^	0.000^b^	0.000^b^
*β_AA_*	156.63	148.71	156.29	143.16	143.53
*β_AB_*	140.41	107.89	139.51	143.16^a^	143.53^a^
*β*_BB_	97.52	84.39	96.63	97.74	97.82
Residual variance	237.84	153.88	226.92	249.41	250.94
Major gene heritability (*h*^2^)	—	—	—	—	0.673
*λ* (Box–Cox parameter)	0.55	0.09	0.49	0.46	0.55
Residual *ρ*_po_=*ρ*_ss_	[0.000]	[0.000]	[0.000]	[0.000]	—
Gender cov	2.79	7.26	2.74	3.10	—
Number of parameters	9	8	8	7	5
−2 ln *L*	765.02	776.23	766.37	767.46	767.92
AIC	783.02	792.23	782.37	781.46	777.92
*P-*value		0.001^c^	0.245^c^	0.296^d^	0.794^e^

*q*_A_=frequency of disease allele; *ψ*=genotype frequencies; *τ*=transmission probability of allele A; *β*=genotype dependent mean; AIC=Akaike information criterion; *ρ*_po_=correlation between parent–offspring pairs; *ρ*_ss_=correlation between siblings. Values in brackets indicate that the estimate reached its boundary.

a Dependent parameter.

b Parameter is fixed to the shown value.

c Likelihood ratio test compared with the general model.

d Compared with the Mendelian model.

e Compared with the autosomal dominant model.
